# Sulfur Metabolism Pathways in *Sulfobacillus acidophilus* TPY, A Gram-Positive Moderate Thermoacidophile from a Hydrothermal Vent

**DOI:** 10.3389/fmicb.2016.01861

**Published:** 2016-11-18

**Authors:** Wenbin Guo, Huijun Zhang, Wengen Zhou, Yuguang Wang, Hongbo Zhou, Xinhua Chen

**Affiliations:** ^1^Key Laboratory of Marine Biogenetic Resources, Third Institute of Oceanography, State Oceanic AdministrationXiamen, China; ^2^Department of Bioengineering, School of Minerals Processing and Bioengineering, Central South UniversityChangsha, China; ^3^Laboratory for Marine Biology and Biotechnology, Qingdao National Laboratory forMarine Science and TechnologyQingdao, China

**Keywords:** sulfur metabolism, *Sulfobacillus acidophilus* TPY, moderate thermoacidophile, transcriptomic analysis, SOR

## Abstract

*Sulfobacillus acidophilus* TPY, isolated from a hydrothermal vent in the Pacific Ocean, is a moderately thermoacidophilic Gram-positive bacterium that can oxidize ferrous iron or sulfur compounds to obtain energy. In this study, comparative transcriptomic analyses of *S. acidophilus* TPY were performed under different redox conditions. Based on these results, pathways involved in sulfur metabolism were proposed. Additional evidence was obtained by analyzing mRNA abundance of selected genes involved in the sulfur metabolism of sulfur oxygenase reductase (SOR)-overexpressed *S. acidophilus* TPY recombinant under different redox conditions. Comparative transcriptomic analyses of *S. acidophilus* TPY cultured in the presence of ferrous sulfate (FeSO_4_) or elemental sulfur (S^0^) were employed to detect differentially transcribed genes and operons involved in sulfur metabolism. The mRNA abundances of genes involved in sulfur metabolism decreased in cultures containing elemental sulfur, as opposed to cultures in which FeSO_4_ was present where an increase in the expression of sulfur metabolism genes, particularly sulfite reductase (SiR) involved in the dissimilatory sulfate reduction, was observed. SOR, whose mRNA abundance increased in S^0^ culture, may play an important role in the initial sulfur oxidation. In order to confirm the pathways, SOR overexpression in *S. acidophilus* TPY and subsequent mRNA abundance analysis of sulfur metabolism-related genes were carried out. Conjugation-based transformation of pTrc99A derived plasmid from heterotrophic *E. coli* to facultative autotrophic *S. acidophilus* TPY was developed in this study. Transconjugation between *E. coli* and *S. acidophilus* was performed on modified solid 2:2 medium at pH 4.8 and 37°C for 72 h. The SOR-overexpressed recombinant *S. acidophilus* TPY-SOR had a ${\text{SO}}_{4}^{2-}$-accumulation increase, higher oxidation/ reduction potentials (ORPs) and lower pH compared with the wild type strain in the late growth stage of S^0^ culture condition. The transcript level of *sor* gene in the recombinant strain increased in both S^0^ and FeSO_4_ culture conditions, which influenced the transcription of other genes in the proposed sulfur metabolism pathways. Overall, these results expand our understanding of sulfur metabolism within the *Sulfobacillus* genus and provide a successful gene-manipulation method.

## Introduction

Bioleaching is the extraction of metals from their ores through the use of acidophilic chemolithotrophic microorganisms (Johnson et al., 2012). The industrial application of bioleaching microorganisms to recover metals from minerals has been well-established (Torma, 1983; Acevedo, 2000; Suzuki, 2001; Rawlings, 2002; Olson et al., 2003). The bioleaching microorganisms have several physiological features in common (Brune and Bayer, 2012). They are all chemolithoautotrophic and are able to use ferrous iron, elemental sulfur or reduced inorganic sulfur compounds (RISCs) as electron donors. According to the temperature at which they grow, bioleaching bacteria can be separated into mesophiles, moderate thermophiles, and thermophiles. Mesophilic microorganisms have been applied successfully for the bioleaching of gold, copper, zinc, and uranium (Brierley and Brierley, 2001; Merroun et al., 2003; Rawlings et al., 2003). However, the rate of bioleaching is limited, partially due to the fact that the bioleaching microorganisms cannot adapt to the complicated leaching conditions such as high concentrations of heavy metals and high temperature. Many researchers have studied the use of thermophilic instead of mesophilic microorganisms to improve the bioleaching rate (Brierley, 1990). However, the majority of extreme thermophiles growing above 60°C are classified as archaea, which always lack typical cell wall and cannot survive in high pulp density of heavy metals due to strong stirring shear (Rawlings, 2002; Rawlings et al., 2003). Leaching sulfide mines with Gram-positive moderately thermophilic bacteria not only dramatically improves the bioleaching rate but also avoids inhibition by high concentration of heavy metals during bioleaching (Robertson et al., 2002; Zhou et al., 2009). Therefore, compared with mesophilic microorganisms and extreme thermophilic microorganisms, the moderately thermophilic microorganisms may offer some advantages in industrial applications of bioleaching (Brierley and Brierley, 2001; Robertson et al., 2002; Zhou et al., 2009). The genus *Sulfobacillus* are moderately thermophilic (40°−60°C), endospore-forming, Gram-positive bacteria that have been isolated from heaps of mineral waste and biomining operations. These bacteria are able to grow autotrophically or heterotrophically. When growing autotrophically, they use ferrous iron, RISCs, or sulfide minerals as electron donors (Norris et al., 1996).

Bioleaching is based upon biological oxidation of iron and RISCs. The model for iron and RISCs oxidation and electron transport has been described in detail in the mesophilic bacterium *Acidithiobacillus ferrooxidans* ATCC 23270 and the extreme thermophilic archaeon *Metallosphaera sedula* DSM 5348 (Auernik et al., 2008; Valdes et al., 2008; Quatrini et al., 2009). The oxidation and electron transfer pathways for RISCs are more complex than those for iron, making their prediction, and elucidation more difficult. Due to the difficulties in developing genetic techniques in acidophiles, a large proportion of the hypotheses regarding RISCs metabolic pathways in these prokaryotes are based on systems biology (Dopson and Johnson, 2012). RISCs oxidation pathways in *A. ferrooxidans* ATCC 23270 are predicted to involve various enzymes, enzyme complexes, and a number of electron carriers located in different cellular compartments (Quatrini et al., 2009). A model of sulfur oxidation in *A. ferrooxidans* ATCC 23270 was proposed, in which electrons from oxidation of RISCs are transferred via the quinol pool (QH_2_) to terminal oxidases to produce ATP, or to NADH complex I to generate NAD(P)H, coupling RISCs oxidation with the generation of energy or reducing power (Quatrini et al., 2009). An integrated sulfur oxidation model that includes various sulfur oxidation pathways was proposed in *A. caldus*, a Gram-negative, acidophilic, obligately chemolithotrophic, moderately thermophilic bacterium (Mangold et al., 2011; Chen et al., 2012). Recently, the sulfur oxidation model of *Sulfobacillus thermosulfidooxidans* was proposed by two independent works via comparative genome analysis (Guo et al., 2014; Justice et al., 2014). Compared with data available for Gram-negative *A. ferrooxidans* and *A. caldus* and Gram-positive *Sulfobacillus thermosulfidooxidans*, to the best of our knowledge, little is known about RISCs oxidation and electron transport mechanisms in moderately thermophilic, Gram-positive *S. acidophilus*. The complexity of the sulfur metabolism system of *S. acidophilus*, as well as the lack of genetic manipulation methods for construction of mutants, represent considerable obstacles to investigation of mechanisms of *S. acidophilus* sulfur metabolism.

Until now, genetic modification of bioleaching microorganisms has been limited, in part, by technical difficulties associated with growing and manipulating these bacteria and, in part, because of public sensitivity to the use of genetically modified organisms (Rawlings, 2002). Genetic transfer between *E. coli* and Gram-negative *A. ferrooxidans* was first reported by Peng et al. (1994). Much effort has been made on the transformation of plasmid to *Sulfobacilli*, but no transformant was obtained (Joubert, 2008). To date, little progress has been made toward the development of genetic systems for the genus *Sulfobacillus*.

In the present study, in order to understand the sulfur metabolism of *S. acidophilus* TPY facilitating its use in bioleaching of minerals in the future, comparative transcriptomic analyses were carried out in the presence of ferrous sulfate (FeSO_4_) or element sulfur (S^0^) to gain global insights into the sulfur metabolism pathways and electron transport in *S. acidophilus* TPY. Then, the huge differences in culture conditions between heterotrophic *E. coli* and facultative autotrophic *S. acidophilus* TPY was overcome. Conjugation based transformation of plasmid to *S. acidophilus* TPY was also developed in this study. Further, the sulfur metabolism pathways were proposed and confirmed by pathway validation of an SOR-overexpressing *S. acidophilus* TPY recombinant. This is the first attempt to characterize the sulfur metabolism pathways of Gram-positive *S. acidophilus* and also the first report of genetic manipulation of the Gram-positive moderate thermoacidophile.

## Materials and methods

### Bacterial strains and growth conditions

Bacterial strains and plasmids used in this study are listed in Supplementary Table 1. *S. acidophilus* TPY was isolated from a hydrothermal vent in the Pacific Ocean (12°42′29″ N, 104°02′01″ W; water depth, 3083 m; Li et al., 2011). It had been deposited in the China Center for Type Culture Collection (CCTCC) with accession number CCTCC M 2010203 (Li et al., 2011). It is a Gram positive bacterium and 0.3~0.5 × 1~3 μm in shape. This strain has the ability to oxidize elemental sulfur and ferrous ion as electron donors. It was grown aerobically on SA medium composed of 3 g/L (NH_4_)_2_SO_4_, 0.5 g/L K_2_HPO_4_, 0.5 g/L MgSO_4_, 0.1 g/L KCl, 0.01 g/L Ca(NO_3_)_2_, 0.2 g/L yeast extract, and 13.9 g/L FeSO_4_ 7H_2_O or 1% (wt/vol) elemental sulfur as the energy source. The initial pH of the medium was adjusted to 1.8 with 2 M H_2_SO_4_. Cultivation was carried out in 250 mL flasks containing 100 mL SA medium on a shaker at 180 rpm and 50°C. *Escherichia coli* strain JM109 was used as the host for plasmid construction, and *E. coli* S17-1 (Simon et al., 1983) as vector donor in conjugation. The *E. coli* strains were grown at 37°C in Luria-Bertani (LB) medium or on LB agar plates supplemented with 100 μg/mL ampicillin, if necessary.

### RNA purification and RNA-Seq

*S. acidophilus* TPY was cultured in the presence of ferrous sulfate or elemental sulfur as the energy source. Cells of 100 mL culture in the late exponential phase of growth, 24 h in ferrous sulfate and 3 days in elemental sulfur culture, were harvested by centrifugation at 6000 × g and 4°C and then washed three times with diluted H_2_SO_4_, pH 2.0 (Supplementary Figure 1). The centrifugation precipitates of the cultures and the diluted H_2_SO_4_ were both placed on ice. Total RNA was isolated from cells using the Trizol Reagent (Invitrogen, Carlsbad, CA, USA) according to the manufacturer's instructions. Only one biological replicate was used in the preparation of RNA samples from each FeSO_4_ and S^0^ cultures. Residual genomic DNA was digested with RNase-free DNase I (TaKaRa, Shiga, Japan). The integrity of each RNA sample was assessed by electrophoresis through a 1.2% agarose gel in 90 mM Tris-boric acid containing 2 mM EDTA (TBE). RNA concentration and purity were determined spectrophotometrically by measuring *A*_260_ and *A*_260_/*A*_280_ ratio. RNA-Seq and subsequent bioinformatics analysis were carried out by Beijing Genomics Institute (BGI) at Shenzhen, China (Liu et al., 2015). The mRNA purification and fragmentation, double-stranded cDNA synthesis, RNA-seq library preparation were carried out as described previously (Qin et al., 2014). The Illumina HiSeq™ 2000 platform was applied for the sequencing (Liu et al., 2015). Reads on which all following analysis are based were collected from sequence data passing BGI's quality control. Sequencing quality assessment including alignment statistics, sequencing randomness assessment and distribution of reads in reference genome (*S. acidophilus* TPY, GenBank accession: CP002901) were carried out (Li et al., 2009). Reads were mapped to reference genome using SOAP2 (Li et al., 2009). The RNA-Seq reads have been deposited in GenBank with accession number SRP055734.

### Identification of differentially transcribed genes

Differentially transcribed genes were identified using a rigorous algorithm developed by BGI based on the method described previously (Audic and Claverie, 1997). The calculation of unigene transcription uses the RPKM (reads per kb per million reads) method (Mortazavi et al., 2008). The calculated gene transcription profile can be used to directly compare gene transcription levels between samples. Genes with log_2_ (ratio RPKM) values >2.0 or < −2.0 were considered to be increased or decreased, respectively. In addition, those genes with false discovery rate (FDR) <0.001 in the samples were also included.

### Total RNA extraction and RT-qPCR assays

In order to verify the transcription levels of genes involved in the sulfur metabolism pathways, RT-qPCR assays were carried out. *S. acidophilus* TPY cultivation and total RNA extraction were conducted as described above for RNA-Seq. Reverse transcription was performed using cDNA synthesis kit (M-MLV Version, TaKaRa) according to the manufacturer's instructions. Transcription levels of representative genes in the sulfur metabolism pathways of *S. acidophilus* TPY were characterized using an ABI PRISM 7500 Real-Time System with a SYBR Green-based assay. Primers used to amplify representative genes of the sulfur metabolism pathways and the 16S rRNA gene, which served as an internal control, are shown in Supplementary Table 2. Total RNA was extracted from three independent cultures with ferrous sulfate or elemental sulfur, respectively. RT-qPCR quantification was performed three times for each RNA sample. Therefore, transcript levels were measured in triplicate for each RNA isolate. The Reverse transcription reaction mixture (20 μL) in a 200 μL tube contained 1 mg Total RNA, 2 μL random primers (Promega, 10 μM), 4 μL 5 × M-MLV Buffer, 1 μL dNTP Mixture, 0.5 μL RNase Inhibitor (40 U/μL), 0.5 μL Reverse Transcriptase M-MLV (RNase H−; 200 UμL) and 12 μL RNase Free water. The reaction was performed at 42°C for 1 h and then at 70°C to end the reaction. Each RT-qPCR mixture (20 μL) in a 200 μL tube contained 10 μL SYBR® Premix Ex Taq™ (Taq DNA polymerase, dNTPs, MgCl_2_, SYBR Green I dye, 2 ×), 0.4 μL PCR Forward Primer (10 μM), 0.4 μL PCR Reverse Primer (10 μM), 1.0 μL SS DNA template, and 8.2 μL H_2_O. The RT-qPCR reaction was performed with the following cycling condition: (1) 50°C for 2 min; (2) 95°C for 3 min; (3) 40 cycles of 95°C for 20 s and 56°C for 40 s and 72°C for 45 s; (4) 4°C hold with data collection at each annealing step. The 16S rDNA was used as the reference gene for normalization. The relative transcription was calculated using the comparative ΔΔCT method (Livak and Schmittgen, 2001). Levels of transcripts in *S. acidophilus* TPY cultivated in FeSO_4_ are expressed as n-fold relative to that of the same gene in *S. acidophilus* TPY cultured in S^0^.

### Construction of sulfur oxygenase reductase (SOR) expression plasmid

As *S. acidophilus* TPY is a Gram-positive bacterium, the *Bacillus E. coli* shuttle vector pTrc99A (Amann et al., 1988) was used to construct the expression plasmid pTrc99A_sor_oriT. Two pairs of primers were designed to amplify the *sor* gene encoding the sulfur oxygenase reductase (SOR) from *S. acidophilus* TPY genomic DNA and *oriT* fragment of plasmid pEx18Tc (Hoang et al., 1998), respectively (Supplementary Table 2). The amplified *sor* fragment was purified and cloned into the *Eco*RI/*Bam*HI site of pTrc99A (Amann et al., 1988) in frame with a 6 × His tag. The resulting plasmid was named pTrc99A_sor. The amplified *oriT* fragment was then cloned into the *Bam*HI/*Hin*dIII site of pTrc99A_sor to form expression vector pTrc99A_sor_oriT (Supplementary Figure 2). *E. coli* S17-1 (Simon et al., 1983) was transformed with the plasmid pTrc99A_sor_oriT for transconjugation, which was carried out using *S. acidophilus* TPY as the recipient bacterium.

### Transconjugation between *E. coli* and *S. acidophilus*

Modified solid 2:2 medium was used as transconjugation medium and prepared in three parts (Peng et al., 1994). Na_2_S_2_O_3_·5H_2_O (2 g) was added to 20 mL of H_2_O (solution A); (NH_4_)_2_SO_4_ (4.5 g), KCl (0.15 g), MgSO_4_·7H_2_O (0.75 g) and yeast extract (0.5 g) were dissolved in 500 mL of H_2_O and then adjusted to pH 4.8 with 2 M H_2_SO_4_ (solution B); Gellan Gum (10 g; Sigma-Aldrich Corporation, USA) was added to 480 mL of H_2_O (solution C). Solutions A was filter sterilized, while solutions B and C were autoclaved. Solutions A, B, and C were mixed together when solutions B and C were cooled to 80°C. The final pH of the medium was 4.8. About 30 mL of modified solid 2:2 medium was poured into each 9 cm-diameter plate. Transconjugation of plasmid pTrc99A_sor_oriT from *E. coli* S17-1 to *S. acidophilus* TPY was conducted by filter mating. Donor cells were harvested by centrifugation at the late exponential growth phase; recipient cells were harvested at the stationary phase. Iron or sulfur precipitates were removed by low-speed centrifugation (100 × g) from SA medium liquid culture. Both the donor and recipient cells were washed three times with elution solution (4.5 g/L (NH_4_)_2_SO_4_, 0.15 g/L KCl, 0.75 g/L MgSO_4_·7H_2_O, pH 4.8) and then mixed at a donor-to-recipient ratio of 1:1. Cell suspension (100 μL) was then transferred to a filter membrane (0.45 μm pore size; 25 mm diameter) placed on the modified solid 2:2 medium. After incubation at 37°C for 72 h, the bacterial lawn on the filter was washed with 3.0 mL of elution solution. The bacterial eluent was diluted and spread on the modified solid 2:2 medium selection plates with 100 μg/mL of ampicillin added to select transconjugants at 50°C.

### Western blot analysis of overexpressed SOR

*S. acidophilus* TPY and recombinant *S. acidophilus* TPY-SOR cells were grown to the late exponential phase after cultivation in 250 mL flasks containing 100 mL of SA medium in the presence of FeSO_4_ as the energy source. Cells were harvested from 50 mL of SA medium by centrifugation at 6000 × g at 4°C, washed three times with diluted H_2_SO_4_, pH 2.0, resuspended in 1 mL PBS buffer (pH 7.4). Then, 20 μL of the cell suspension was mixed with 80 μL 5 × SDS-PAGE loading buffer and boiled at 100°C for 10 min. The total protein extracts sample (10 μL) from the *S. acidophilus* TPY and recombinant *S. acidophilus* TPY-SOR were separated by SDS-PAGE through 15% acrylamide gels, and electrotransferred onto polyvinylidene difluoride (PVDF) membranes using standard methods. The PVDF membrane was then washed with Tris-buffered saline containing 0.1% Tween 20 (TBST) twice for 5 min each. The membrane was then incubated in anti-6 × His tag monoclonal antibody (Roche) as the primary antibody and then horseradish peroxidase-conjugated polyclonal rabbit anti-mouse IgG (Thermo Scientific, Waltham, MA, USA) as the secondary antibody. Immunoreactive proteins were detected using NBT/BCIP (Thermo Scientific) as substrate according to the manufacturer's recommendations.

### Characterization of recombinant strain *S. acidophilus* TPY-SOR

Equal amounts of inoculum (5%, v/v) of *S. acidophilus* TPY and recombinant *S. acidophilus* TPY-SOR were inoculated into 250 mL flasks containing 100 mL of SA medium with 1% (w/v) elemental sulfur as energy source. Flasks were incubated at 50°C and 200 rpm on a rotary shaker for 11 days. Variations in ORPs, pH, and ${\text{SO}}_{4}^{2-}$ concentration of the cultures were measured using an ORP meter, pH meter, and BaSO_4_ turbidimetry (Agterdenbos and Martinius, 1964). *S. acidophilus* TPY and *S. acidophilus* TPY-SOR were compared in the three parameters above when they were cultivated under the same conditions. The *S. acidophilus* TPY and *S. acidophilus* TPY-SOR cultivation were done in triplicate and the results are shown as the mean ± *SD*. Meanwhile, *S. acidophilus* TPY and *S. acidophilus* TPY-SOR cells were grown to the late exponential phase after inoculation in SA medium in the presence of FeSO_4_ or S^0^ as the energy source. RT-qPCR was carried out to analyse levels of transcripts of representative genes in the sulfur metabolism pathways in the recombinant *S. acidophilus* TPY-SOR under FeSO_4_ or S^0^ cultivation condition, respectively, using the wild type strain *S. acidophilus* TPY as control.

## Results

### General features of the transcriptional profiles generated by RNA-Seq and sulfur metabolism pathways of *S. acidophilus* TPY

**Figure 1:**
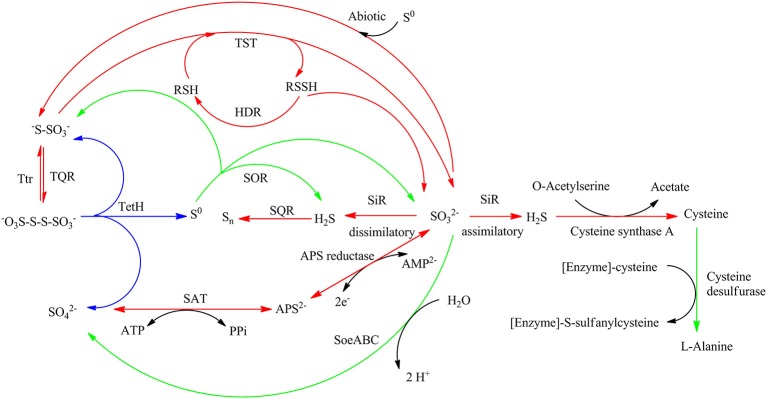
**Proposed sulfur metabolism pathways of ***Sulfobacillus acidophilus*** TPY**. Arrows in red and green indicate reactions carried out by genes or operons with mRNA abundance increased under conditions of growth in FeSO_4_ and S^0^, respectively. Arrows in blue indicate reactions carried out by genes or operons with mRNA abundance almost the same under conditions of growth in FeSO_4_ and S^0^. SQR, sulfide quinone reductase; TQR, thiosulfate quinone reductase; TetH, tetrathionate reductase; TST, thiosulfate sulfur transferase (rhodanese); HDR, heterodisulfide reductase; APS reductase, adenosine phosphosulfate reductase; SAT, sulfate adenylate transferase (sulfate adenylyltransferase, ATP sulfurylase); SiR, sulfite reductase; Ttr, tetrathionate reductase; SOR, sulfur oxygenase reductase; Psr, polysulfide reductase.

Comparative analysis of the transcribed gene profiles has provided extensive biological information about the response of *S. acidophilus* TPY grown in the presence of S^0^ or FeSO_4_ on a genomic scale. Using statistical criteria described previously, a 2.0 log_2_ (ratio RPKM) of median cutoff was considered as differential gene transcription under the two growth conditions (Mortazavi et al., 2008). A total of 841 genes showed a differential transcription profile, of which 507 were increased (by up to 14.4-fold) and 334 were decreased (by up to 13.5-fold) in FeSO_4_ compared to S^0^ (Supplementary Table 3). Genes exhibiting differential transcription were annotated by Gene Ontology and KEGG and were found to be mostly associated with unknown functions, energy metabolism and central intermediary processes. Genes and operons involved in the sulfur metabolism pathways were analyzed in further detail according to their differential mRNA abundance levels (Table 1). Quantitative reverse transcription PCR analysis of representative genes involved in the sulfur metabolism confirmed the transcriptome results (Table 1). The RNA samples analyzed for the two methods were different, strongly suggesting that the biological observations were reproducible. Reactions and related genes or operons involved in the sulfur metabolism *of S. acidophilus* TPY were listed in Table 2. Based on these reactions, we proposed the *S. acidophilus* TPY sulfur metabolism pathways with redox cycles shown in Figure 1.

**Table 1:** **Gene clusters involved in the sulfur metabolism and electron transfer of ***Sulfobacillus acidophilus*** TPY**.

	**Gene**	**Function**	**S-RPKM^a^**	**FeSO_4_-RPKM^b^**	**log_2_ (FeSO_4_/S)^c^**	**FDR^d^**	**RT-PCR^e^**
**PET I OPERON**							
TPY_3079	*petC-1*	Cytochrome c subunit of the bc complex	471.4	6028.6	3.68	0	4.92±0.22
TPY_3080	*petB-1*	Cytochrome b subunit of the bc complex	150.2	4771.0	4.99	3.1 × 10^−13^	
TPY_3081		Uncharacterized protein required for cytochrome oxidase assembly	191.8	136.5	−0.49	2.94 × 10^−14^	
**CYTOCHROME** ***bd*** **UBIQUINOL OXIDASE**							
TPY_1817	*cydA*	Cytochrome *bd* ubiquinol oxidase subunit I	148.8	99.4	−0.58	2.09 × 10^−21^	
TPY_1843			5.3	24.1	2.17	1.88 × 10^−13^	
TPY_3556			11.3	9.8	−0.21	3.7 × 10^−8^	
TPY_1818	*cydB*	Cytochrome d ubiquinol oxidase, subunit II	155.1	83.3	−0.90	2.13 × 10^−33^	
TPY_1842			33.6	57.7	0.78	2.1 × 10^−10^	
TPY_3557			17.4	7.7	−1.17	8.05 × 10^−7^	
**CYTOCHROME** ***bo***_3_ **UBIQUINOL OXIDASE**							
TPY_0367	*cyoD*	Cytochrome o ubiquinol oxidase, subunit IV	177.6	50.0	−1.83	1.08 × 10^−43^	
TPY_0366	*cyoC*	Cytochrome o ubiquinol oxidase, subunit III	512.9	143.9	−1.83	1.91 × 10^−210^	
TPY_0365	*cyoB*	Cytochrome o ubiquinol oxidase, subunit I	741.3	160.3	−2.21	0	
TPY_0364	*cyoA*	Cytochrome o ubiquinol oxidase, subunit II	527.7	78.0	−2.76	0	0.14±0.02
**SULFATE REDUCING OPERON**							
TPY_2305	*cysI*	Sulfite reductase (NADPH) hemoprotein beta-component	4.8	2522.7	9.03	1.36 × 10^−13^	2.31±0.24
TPY_2304	*cysH*	Adenosine phosphosulfate (APS) reductase	3.7	1141.8	8.27	2.74 × 10^−8^	2.17±0.18
TPY_2303	*sat*	Sulfate adenylyltransferase	3.2	1410.7	8.79	6.65 × 10^−10^	1.51±0.11
TPY_2302	*cysG*	Siroheme synthase (precorrin-2 oxidase/ferrochelatase domain)	0.8	648.4	9.67	1.79 × 10^−14^	
TPY_2301	*cysG*	Uroporphyrin-III C-methyltransferase	5.5	824.2	7.21	3.53 × 10^−11^	
TPY_2300	*ahpC*	Peroxiredoxin (alkyl hydroperoxide reductase subunit C)	35.0	1915.0	5.78	6.4 × 10^−13^	
TPY_2299		Hypothetical protein new	0^f^	20.7	14.34	4.25 × 10^−6^	
**THIOSULFATE-QUINONE OXIDOREDUCTASE COMPLEX OPERON**							
TPY_2169	d*oxDA*	Thiosulfate-quinone oxidoreductase small subunit DoxD	1.5	99.3	6.08	3.07 × 10^−5^	2.22±0.20
TPY_2170		Periplasmic solute-binding protein, putative	3.7	293.9	6.30	2.45 × 10^−7^	
TPY_2171		Tat (twin-arginine translocation) pathway signal sequence domain protein	1.9	165.1	6.41	1.79 × 10^−4^	
TPY_2172		C4-dicarboxylate transporter/malic acid transport protein	2.7	35.4	3.71	8.22 × 10^−6^	
**TETRATHIONATE HYDROLASE**							
TPY_0895	*tetH*	Tetrathionate hydrolase	12.2	15.0	0.30	0.089	1.34±0.15
**TETRATHIONATE REDUCTASE OPERON**							
TPY_3698	*tatA*	Sec-independent protein translocase protein TatA	5.8	17660.1	11.6	4.14 × 10^−6^	
TPY_3699	*ttrB*	Tetrathionate reductase subunit B	7.2	3821.3	9.06	3.49 × 10^−13^	
TPY_3700	*ttrC*	Tetrathionate reductase subunit C	4.6	871.0	7.57	4.68 × 10^−13^	1.80±0.15
TPY_3701	*ttrA*	Molybdopterin dinucleotide-binding region	9.0	838.6	6.54	8.27 × 10^−13^	
TPY_3702	*ttrD*	Tetrathionate reductase subunit D	31.8	772.7	4.60	5.76 × 10^−13^	
TPY_3703	*tatC*	Twin arginine-targeting protein translocase TatC	27.3	841.3	4.94	2.07 × 10^−13^	
**SULFIDE-QUINONE OXIDOREDUCTASE**							
TPY_3704	*sqr*	Sulfide-quinone oxidoreductase	83.9	246.6	1.56	0	
TPY_3731			1.0	1.5	0.65	4.2 × 10^−7^	
**SULFUR OXYGENASE REDUCTASE**							
TPY_0405	*sor*	Sulfur oxygenase reductase	44.6	4.5	−3.32	1.41 × 10^−53^	0.33±0.27
**SoeABC**							
TPY_0113	*SoeA*	Molybdopterin oxidoreductase, molybdopterin binding subunit	319.4	6.8	−5.55	0	
TPY_0114	*SoeB*	Molybdopterin oxidoreductase, iron-sulfur binding subunit	504.9	14.3	−5.14	0	
TPY_0115	*SoeC*	Molybdopterin oxidoreductase subunit C, membrane anchor subunit	499.2	25.7	−4.28	0	0.47±0.11
**HETERODISULFIDE REDUCTASE COMPLEX OPERON**							
TPY_3532	*dsrE*	Hypothetical protein	15.3	121.9	3.00	3.5 × 10^−13^	
TPY_3531	*hdrC*	Heterodisulfide reductase, subunit C	27.3	95.5	1.80	0	
TPY_3530	*hdrB*	Heterodisulfide reductase, subunit B	13.0	46.7	1.84	2.69 × 10^−13^	
TPY_3529	*hdrA*	Heterodisulfide reductase, subunit A, and related polyferredoxins	10.8	27.7	1.35	1.10 × 10^−12^	
TPY_3528	*orf2*	Hypothetical protein	3.3	11.9	1.84	3.69 × 10^−6^	
TPY_3527	*hdrD*	heterodisulfide reductase subunit D	12.8	36.3	1.50	0	
**RHODANESE**							
TPY_2911	*rhd*	Rhodanese-related sulfurtransferase	766.2	73.8	−3.38	0	
TPY_0056	*tusA*	SirA family protein, Rhodanese-related sulfurtransferase	18.1	486.1	4.75	1.74 × 10^−11^	
TPY_1113			825.8	172.0	−2.26	8.36 × 10^−163^	
TPY_3523			2.1	5.5	1.38	0.195	
TPY_3767			69.2	48.8	−0.50	0.032	
TPY_0110			66.4	3.9	−4.09	9.05 × 10^−32^	
**OTHERS**							
TPY_0363	*cysK*	Cysteine synthase A	87.6	1528.3	4.12	6.32 × 10^−13^	
TPY_3319	*sufS*	Cysteine desulfurase	1216.4	203.3	−2.58	0	

a *RPKM-values of genes from S. acidophilus TPY cultured in the presence of elemental sulfur as the energy source. RPKM, reads per kb per million reads*.

b *RPKM-values of genes from S. acidophilus TPY cultured in the presence of ferrous sulfate as the energy source*.

c *The log_2_ ratio of FeSO_4_-RPKM/S-RPKM*.

d *False discovery rate*.

e *Levels of transcripts in S. acidophilus TPY cultivated in FeSO_4_ from RT-PCR are expressed as n-fold relative to that of S. acidoLphilus TPY cultivated in S^0^*.

f *The RPKM-value was set as 0.001 in the calculation of log2 ratio of (FeSO_4_-RPKM/S-RPKM) when its value equals zero*.

**Table 2:** **Reactions of selected enzymes that require inorganic sulfur compounds**.

**NO**.	**Reactions**	**Enzymes**	**Locus of TPY**	**References**
1	n HS^−^ + n quinine → ^−^S-(S)_*n*−2_-S^−^+ n quinol	Sulfide quinone reductase (SQR)	TPY_3704, 3731	Quatrini et al., 2009
2	2 ^−^S-${\text{SO}}_{3}^{-}$+ 2 Ferricytochrome c → ^−^O_3_S-S-S-${\text{SO}}_{3}^{-}$+ 2 Ferrocytochrome c	Thiosulfate quinone oxidoreductase (TQR)	TPY_2169	R00029^a^
3	^−^O_3_S-S-S-${\text{SO}}_{3}^{-}$→^−^S-${\text{SO}}_{3}^{-}$ + ${\text{SO}}_{4}^{2-}$+ S^0^	Tetrathionate hydrolase (TetH)	TPY_0895	De Jong Gah et al., 1997
4	4S^0^ + 4 H_2_O + O_2_ → 2 H_2_S + 2 H${\text{SO}}_{3}^{-}$+ 2 H^+^	Sulfur oxygenase reductase (SOR)	TPY_0405	Chen et al., 2012, R07365
5	RSH + ^−^S-${\text{SO}}_{3}^{-}$ → RSSH + ${\text{HSO}}_{3}^{-}$	Thiosulfate sulfur transferase (TST, rhodanese)	TPY_0056, 0110, 1113, 2911, 3523, 3767	Chen et al., 2012
6	RSSH → RSH + ${\text{HSO}}_{3}^{-}$	Heterodisulfide reductase (HDR)	TPY_3527-3532	Chen et al., 2012
7	AMP + ${\text{HSO}}_{3}^{-}$+ Glutathione disulfide < = > Adenylyl sulfate + 2 Glutathione	Adenosine phosphosulfate (APS) reductase	TPY_2304	R05717
8	Adenylyl sulfate + Diphosphate < = > ATP + H_2_SO_4_	Sulfate adenylyltransferase (SAT)	TPY_2303	R00529
9	${\text{HSO}}_{3}^{-}$+ 3 NADPH + 3H^+^ → 3 NADP + 3 H_2_O + HS^−^	Sulfite reductase (SiR)	TPY_2305	R00858, Zeghouf et al., 2000
10	^−^O_3_S-S-S-${\text{SO}}_{3}^{-}$+ 2e- → 2 ^−^S-${\text{SO}}_{3}^{-}$	Tetathionate reductase (Ttr)	TPY_3698–3703	Hensel et al., 1999
11	H_2_SO_3_ + H_2_O → H_2_SO_4_ + 2H^+^	molybdopterin binding oxidoreductase; SoeABC	TPY_0113, 0114, 0115	Dahl et al., 2013, R00533
12	O-Acetyl-L-serine + H_2_S < = > L-Cysteine + Acetate	Cysteine synthase A	TPY_0363	R00897
13	[Enzyme]-cysteine + L-Cysteine < = > [Enzyme]-S-sulfanylcysteine + L-Alanine	Cysteine desulfurase	TPY_3319	R07460, Mihara and Esaki, 2002

a *Reaction number in KEGG database (http://www.genome.jp/kegg/)*.

### Genes in the sulfate reducing operon with mRNA abundance increased under conditions of growth in FeSo_4_

It can be clearly seen from Table 1 that genes in the sulfate reducing operon were increased by as much as 14.34-fold under conditions of growth in FeSO_4_. Reduction of sulfate to sulfide can be divided into two steps: (i) reduction of sulfate to sulfite (Table 2, reactions 7 and 8), which is associated with conversion of ATP to AMP and pyrophosphate via sulfate adenylyltransferase encoded by *sat* (TPY_2303, 8.79-fold increase in FeSO_4_) and adenosine phosphosulfate (APS) reductase encoded by *cysH* (TPY_2304, 8.27-fold increase in FeSO_4_), and (ii) the six-electron reduction of sulfite to sulfide, which is carried out by the sulfite reductase (SiR, Table 2, reaction 9) encoded by *cysI* (TPY_2305, 9.03-fold increase in FeSO_4_, Table 1). The other genes (TPY_2299–2302) in the sulfate reducing operon that encode enzymes involved in the synthesis of siroheme were also increased 5.78- to 14.34-fold under conditions of growth in FeSO_4_. Siroheme is a heme-like prosthetic group used by some enzymes to accomplish the six-electron reduction of sulfur (Murphy et al., 1974). It plays a major role in the sulfur metabolism pathways by converting sulfite to a biologically useful sulfide (Thomas and Surdin-Kerjan, 1997).

### Other genes and operons with mRNA abundance increased under conditions of growth in FeSo_4_

Sulfide generated under SiR catalysis in the FeSO_4_-containing medium would be oxidized into polysulfide by the mRNA abundance increased sulfide-quinone oxidoreductase (SQR; Table 2, reaction 1) encoded by *sqr* (TPY_3704, 1.56-fold increase in FeSO_4_, Table 1). Sulfide would also be used during the synthesis of L-cysteine (Zeghouf et al., 2000). As a result, the cysteine synthase A encoding gene *cysK* (TPY_0363) involved in the synthesis of L-cysteine was also increased 4.12-fold under FeSO_4_ growth condition. However, the cysteine desulfurase encoding gene *sufS* (TPY_3319) was decreased 2.58-fold under FeSO_4_ growth condition. Cysteine desulfurase decomposes L-cysteine to L-alanine and sulfane sulfur via the formation of an enzyme bound persulfide intermediate (Mihara and Esaki, 2002). In addition to producing sulfide, another product of sulfite would be thiosulfate in the presence of S^0^ by an abiotic process. In FeSO_4_-containing growth medium, a high mRNA abundance of the thiosulfate/quinone oxidoreductase (TQR) complex operon was observed, which might be due to the deduced accumulation of thiosulfate. TQR, encoded by *doxDA* (TPY_2169, 6.08-fold increase in FeSO_4_, Table 1), catalyzed the conversion of thiosulfate into tetrathionate, and conversely, tetrathionate would be reduced by tetrathionate reductase (Ttr), encoded by *ttrA* (TPY_3701, 6.54-fold increase in FeSO_4_), *ttrB* (TPY_3699, 9.06-fold increase in FeSO_4_), *ttrC* (TPY_3700, 7.57-fold increase in FeSO_4_) and *ttrD* (TPY_3702, 4.60-fold increase in FeSO_4_; Hensel et al., 1999). Also embedded in the tetrathionate reductase operon were *tatA* (TPY_3698, 11.6-fold increase in FeSO_4_) and *tatC* (TPY_3703, 4.94-fold increase in FeSO_4_), which encode twin arginine-targeting protein translocase and may be involved in transportion of TtrB and TtrA across membranes (Berks et al., 2000). Tetrathionate can also be hydrolyzed by tetrathionate hydrolase (TetH) to thiosulfate, sulfate, and S^0^. TetH is encoded by *tetH* (TPY_0895), which was transcribed at almost identical levels in the presence of S^0^ and FeSO_4_. Thiosulfate sulfur transferase (TST), encoded by *tusA* (TPY_0056), was increased 4.75-fold in FeSO_4_. The thiol proteins (RSH) can be used as sulfur atom acceptors for the catalysis of thiosulfate to sulfite by TST, producing sulfane sulfate (RSSH) which is the substrate of the heterodisulfide reductase complex (HDR; Chen et al., 2012). Thus, RSH obtains a sulfur atom to form RSSH catalyzed by TST, and then RSSH is oxidized by HDR to regenerate RSH (Table 2, reactions 5 and 6). With growth in FeSO_4_, mRNA abundances of all six *hdr* genes were increased up to 3.0-fold compared with growth in S^0^ (Table 1). Besides, the *petII* operon, which encodes an important component of the electron transfer system in *A. ferrooxidans* ATCC 23270 (Valdes et al., 2008; Quatrini et al., 2009), was not found in the *S. acidophilus* TPY genome. It has been presumed that the *petI* operon participates not only in the electron transfer of Fe^2+^ oxidation but also that of RISCs oxidation (Table 1).

### Genes and operons with mRNA abundance increased under conditions of growth in S^0^

Although three copies of *cydAB* (TPY_1817–1818, TPY_1843–1842, and TPY_3556–3557) encode *bd* oxidase, only the copy TPY_1817–1818 had a high RPKM-value under conditions of growth in both S^0^ and FeSO_4_, indicating a high level of transcription. This highly transcribed copy of *cydAB*, together with *cyoABCD* (TPY_0364–0367), encoding a *bo*_3_ oxidase, *SoeABC* (TPY_0113–0115) encoding molybdopterin oxidoreductase and *sor* (TPY_0405) encoding sulfur oxygenase reductase (SOR) were increased under conditions of growth in S^0^. SOR has been found to play a central role in the cytoplasmic sulfur oxidation pathways in several acidophilic and thermophilic archaea (Kletzin et al., 2004; Ghosh and Dam, 2009). SOR is able to catalyze the disproportionation of S^0^, producing sulfite, thiosulfate, and sulfide (Table 2, reaction 4). These products then follow into corresponding oxidation pathways transferring electrons to quinone (QH_2_). Electrons produced from the elemental sulfur and RISCs oxidation via QH_2_ were transferred to the terminal oxidases (*bd* and *bo*_3_) and the NADH complex to produce ATP and NAD(P)H, respectively. When *S. acidophilus* TPY was grown in S^0^-containing medium, *sor* was induced directly by S^0^ with 3.32-fold increase. Subsequently, *cydAB* (TPY_1817–1818), encoding *bd* oxidase, and *cyoABCD* (TPY_0364–0367), encoding a *bo*_3_ oxidase were also increased by up to 2.76-fold under conditions of growth in S^0^. Also increased under S^0^ growth conditions were SoeABC encoding genes TPY_0113, 0114, and 0115. SoeABC which consists of an NrfD/PsrC like membrane anchor (SoeC) and two cytoplasmic subunits: an iron-sulfur protein (SoeB) and a molybdoprotein with an N-terminal iron-sulfur cluster binding site (SoeA) was thought to be a major enzyme catalysing direct oxidation of sulfite to sulfate in the cytoplasm of *Allochromatium vinosum* (Dahl et al., 2013). The high mRNA abundance levels of *SoeABC* under S^0^ growth conditions was presumed to be due to the accumulation of sulfite derived from the product of SOR.

### Overexpression of *sor* in *S. acidophilus* TPY

**Figure 2:**
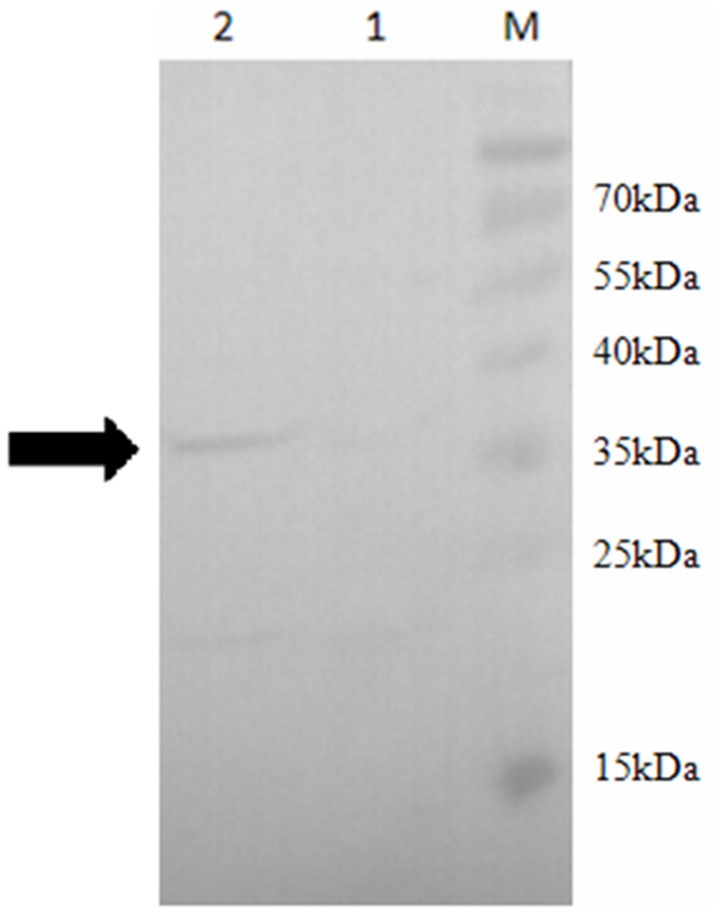
**Western blot of total protein extract of wild-type strain ***S. acidophilus*** TPY and its recombinant ***S. acidophilus*** TPY-SOR**. Total protein extract of wild-type strain *S. acidophilus* TPY (lane 1) and recombinant strain *S. acidophilus* TPY-SOR (lane 2). Lane M, PageRuler Prestained Protein Ladder (Thermo Scientific).

In order to confirm the role of SOR in the sulfur metabolism pathways in *S. acidophilus* TPY, overexpression of *sor* was performed. Due to its important role in the initial oxidation of S^0^ to sulfate, SOR was chosen as the target enzyme for overexpression in *S. acidophilus* TPY. The SOR expression plasmid pTrc99A_sor_oriT was successfully transferred from heterotrophic *E. coli* S17-1 to chemoautotrophic *S. acidophilus* TPY via conjugation of these two strains despite their considerable differences in cultivation conditions. Western blot analysis showed a 35 kDa band in the total protein sample of recombinant *S. acidophilus* TPY-SOR (Figure 2, lane 2), indicating that SOR was successfully overexpressed. It was in accordance with the protein size of SOR from *S. acidophilus* TPY reported before (Zhang et al., 2013). A faint band at the position about 22 kDa was present in the total protein sample of recombinant *S. acidophilus* TPY-SOR (Figure 2, lane 2). Searching in the whole 3754 peptide sequences of the *S. acidophilus* TPY (GenBank accession number: CP002901) using BioEdit program with 6 × His tag sequence as the query revealed that the protein SAM-dependent methyltransferase (TPY_0930) with 202 amino acid residues and 22.7 kDa molecule mass possessed a 7 × His sequence.

### Effect of SOR overexpression on *S. acidophilus* TPY-SOR

**Figure 3:**
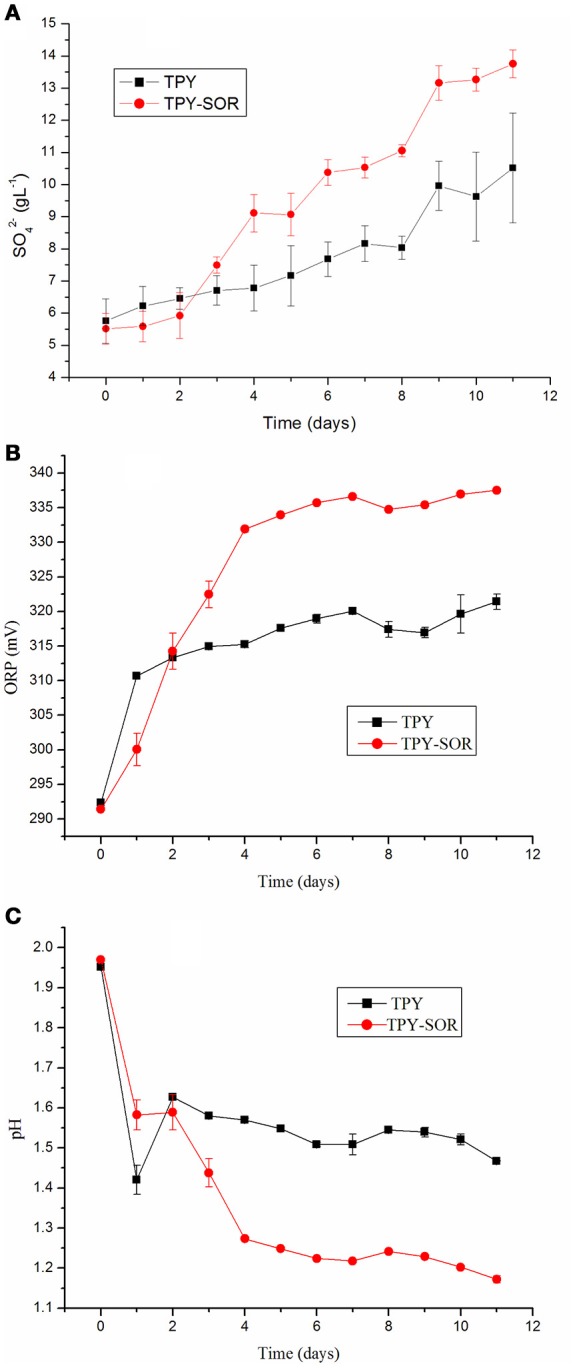
**Characterization of wild-type strain ***S. acidophilus*** TPY and recombinant ***S. acidophilus*** TPY-SOR cultured under the same conditions with elemental sulfur (S^**0**^) as substrate**. ${\text{SO}}_{4}^{2-}$ **(A)**, oxidation/reduction potential (ORP; **B**), and pH **(C)** curves for the wild-type (TPY) and recombinant (TPY-SOR) strains.

*S. acidophilus* TPY and *S. acidophilus* TPY-SOR were cultivated under the same conditions with S^0^ as energy source to investigate changes in pH, oxidation/reduction potentials (ORPs), and amount of ${\text{SO}}_{4}^{2-}$ released into the medium of the recombinant strain. As shown in Figure 3A, when S^0^ was used as the substrate, the recombinant had an obvious ${\text{SO}}_{4}^{2-}$ accumulation increase compared with the wild type in the last 8 days (day 3–11) of cultivation. The maximum ${\text{SO}}_{4}^{2-}$ concentration in the medium was 13.76 g/L, which was 30.8% higher than in the wild-type culture (10.52 g/L). However, the ${\text{SO}}_{4}^{2-}$ concentration in the recombinant culture was slightly less than that in the wild-type in the first 2 days of cultivation. Thus, the two curves cross between day 2 and 3 (Figure 3A). The ORPs curves were similar to the ${\text{SO}}_{4}^{2-}$ concentration curves, where the recombinant ORP-value was higher than that of the wild-type strain after the time point where the curves crossed (Figure 3B). On day 11, the maximum ORP-value of the recombinant was 337.5 mV, which was 5.0% higher than that of the wild-type strain (321.4 mV; Figure 3B). In contrast, the pH curves showed an opposite trend, with recombinant culture having lower pH than the wild type after the curves crossed on day 2 (Figure 3C). On day 11, the minimum pH of the recombinant was 1.17, which was 20.4% lower than the pH of the wild-type culture (1.47; Figure 3C).

### Analysis of mRNA abundance in *S. acidophilus* TPY-SOR

**Figure 4:**
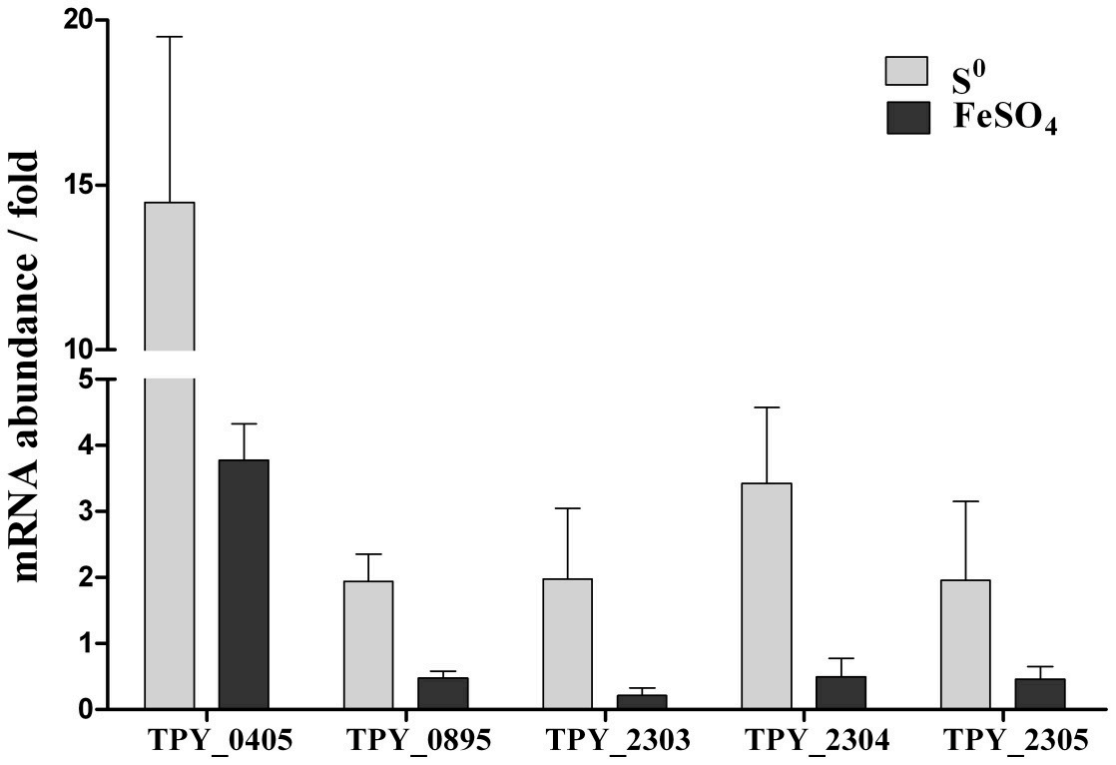
**Effect of energy source on relative mRNA abundance of representative genes in the sulfur metabolism pathways in recombinant ***S. acidophilus*** TPY-SOR**. All values are expressed as n-fold relative to the level of transcripts of the same gene in the wild-type *S. acidophilus* TPY. TPY_0405, *sor* encoding sulfur oxygenase reductase (SOR); TPY_0895, *tetH* encoding tetrathionate hydrolase (TetH); TPY_2303, *sat* encoding sulfate adenylyltransferase (SAT); TPY_2304, *cysH* encoding adenosine phosphosulfate (APS) reductase; TPY_2305, *cysI* encoding sulfite reductase (SiR).

Levels of transcripts of representative genes in the sulfur metabolism pathways in the recombinant *S. acidophilus* TPY-SOR cultured in the presence of S^0^ and FeSO_4_ were analyzed using rRT-qPCR (Figure 4). All values are expressed as n-fold relative to the level of transcripts of the same gene in the wild-type *S. acidophilus* TPY. In the presence of S^0^, the level of transcripts of the *sor* gene (TPY_0405) in *S. acidophilus* TPY-SOR increased by 14.46-fold compared with *S. acidophilus* TPY (Figure 4). In addition to the *sor* gene, the transcription levels of other important genes in the sulfur metabolism pathways also increased; these include *tetH* (TPY_0895), encoding a tetrathionate hydrolase (TetH), with a 1.94-fold increase; *sat* (TPY_2303), encoding sulfate adenylyltransferase (SAT); *cysH* (TPY_2304), encoding adenosine phosphosulfate (APS) reductase; and *cysI* (TPY_2305) encoding sulfite reductase (SiR) in the sulfate reducing operon with increase folds of 1.97, 3.42, and 1.95, respectively (Figure 4). Under conditions of growth in FeSO_4_, the level of *sor* gene (TPY_0405) transcripts in *S. acidophilus* TPY-SOR increased by 3.77-fold compared with *S. acidophilus* TPY (Figure 4). However, the transcription level of other representative genes involved in sulfur metabolism pathways generally decreased, including *tetH* (TPY_0895), *sat* (TPY_2303), *cysH* (TPY_2304), and *cysI* (TPY_2305) with 0.47-, 0.21-, 0.49-, and 0.46-fold decreases, respectively (Figure 4).

## Discussion

### The key role of sulfite reductase (SiR)

In this study, the sulfur metabolism pathways of *S. acidophilus* TPY were proposed based on comparative transcriptomic analyses. The mRNA abundance of sulfur metabolism related genes in S^0^ and FeSO_4_ culture conditions was quite different with that of the type strain *A. ferrooxidans* ATCC 23270. In the type strain *A. ferrooxidans* ATCC 23270, the sulfur metabolism-related genes were significantly increased under conditions of growth in S^0^ and decreased in FeSO_4_ (Quatrini et al., 2009). In addition, in the *A. ferrooxidans* ATCC 23270 genome (GenBank: CP001219), the *cysI* gene encodes a sulfite reductase (NADPH) hemoprotein beta-component with locus tag of AFE_3122 (Zeng et al., 2008). However, in this study, it is interesting that most genes and operons involved in sulfur and RISCs metabolism in *S. acidophilus* TPY were decreased when grown in the presence of S^0^ and increased in the presence of FeSO_4_. In this study, it was speculated that the sulfite reductase (SiR) in *S. acidophilus* TPY may involve in the dissimilatory sulfate reduction and play an important role when FeSO_4_ serves as energy resource (Figure 1). It still could not be excluded as a sulfite reductase (SiR) involving in the assimilatory sulfate reduction (Figure 1). In *E. coli*, the sulfite reductase (SiR) is a 780 kDa soluble complex composed of two proteins, a flavoprotein (SiR-FP) and a metalloprotein (SiR-HP; Zeghouf et al., 2000). In *S. acidophilus* TPY, only the SiR-HP-encoding gene, *cysI* (TPY_2305, 9.03-fold increase in FeSO_4_) was found. The amino acid sequence of sulfite reductase (SiR) of *S. acidophilus* TPY has 34.51% identity with that of type strain *A. ferrooxidans* ATCC 23270. The presence of SiR-HP in *S. acidophilus* TPY would promote the reduction of sulfate to sulfide under conditions of aerobic growth in FeSO_4_. Subsequently, sulfide would be changed into other RISCs. Otherwise, the genes and operons involved in sulfur and RISCs metabolism in *S. acidophilus* TPY would not be increased in the FeSO_4_ cultivation condition. Thus, more ${\text{SO}}_{4}^{2-}$ available in the presence of FeSO_4_ than in the presence of S^0^ increased transcription of genes in the sulfate reducing operon (Table 1). SiR mostly exists in sulfate reducing bacteria (SRB) which usually grow in anaerobic conditions (Wang et al., 2008). However, some SRB are reported to be oxygen tolerant (Kjeldsen et al., 2004). Although most of the genes, enzymes and operons involved in the sulfur metabolism have been reported in *Sulfobacillus thermosulfidooxidans*, the key enzyme SiR which plays an important role in *S. acidophilus* TPY is missing in *S. thermosulfidooxidans* (Guo et al., 2014; Justice et al., 2014). Thus, *S. acidophilus* TPY not only could oxidize RISCs to sulfate ultimately but also reduce sulfate to RISCs, making sulfur redox metabolism a cycle.

### mRNA abundance analysis of *S. acidophilus* TPY-SOR confirmed the proposed sulfur metabolism pathways

The transcription level of *sor* gene in the recombinant *S. acidophilus* TPY-SOR increased by 14.46- and 3.77-fold compared with *S. acidophilus* TPY in the S^0^ and FeSO_4_ culture conditions, respectively. This finding indicates that introduction of extra copies of the *sor* gene under the control of the Trc promoter significantly increased the level of *sor* mRNA in *S. acidophilus* TPY-SOR. The increased ${\text{SO}}_{4}^{2-}$ accumulation in the SOR-overexpressing recombinant compared with the wild type in the late stage of growth (Figure 3A) indicating the increased transportation of S^0^ into cell and transformation of S^0^–${\text{SO}}_{4}^{2-}$. Therefore, the higher fold increase of the recombinant in the S^0^ culture condition indicating the higher transcription of the chromosomal copy of *sor* gene under the native promoter which was induced by higher transported S^0^. Obviously, the extra copies of the *sor* gene under the control of the Trc promoter in the plasmid could not be induced by S^0^. According to the sulfur metabolism pathways proposed above, TetH, SAT, and APS reductase play important roles in the oxidation of RISCs produced from oxidation of sulfur to sulfate. In this study, it was presumed that the overexpression of SOR in *S. acidophilus* TPY-SOR resulted in increase of thiosulfate and sulfite when it was cultivated in the S^0^ containing medium (Figure 1). SOR is able to catalyze the disproportionation of S^0^, producing sulfite, thiosulfate, and sulfide. Thiosulfate and sulfite could be transferred to tetrathionate and APS by TQR and APS reductase, respectively. Subsequently, more tetrathionate and APS accumulated to serve as the substrates of TetH and SAT, respectively, and both produce sulfate (Figure 1). In the S^0^ cultivation condition, the increased transcript levels of the respective genes (*tetH*, TPY_0895; *cysH*, TPY_2304; and *cysI*, TPY_2305) may be one of the reasons for the increased sulfur-oxidation activity of *S. acidophilus* TPY-SOR (Figure 4). This phenomenon was in accordance with the sulfate accumulation increase in *S. acidophilus* TPY-SOR under conditions of growth in S^0^ (Figure 3A). The level of transcripts of the *cysI* gene, encoding SiR, increased by 1.95-fold maybe due to the accumulation of its substrate, sulfite. Under conditions of growth in FeSO_4_, according to the proposed sulfur metabolism pathways (Figure 1), it is presumed that the increased sulfite (one of the products of SOR) accumulation in *S. acidophilus* TPY-SOR simultaneously inhibited the transcription of *sat* and *cysH*, which encode SAT and APS reductase, respectively, participating in the conversion of sulfate to sulfite (Figure 4). Similarly, the deduced thiosulfate accumulation increase in *S. acidophilus* TPY-SOR resulted in decreased transcription of *tetH*, which encodes tetrathionate hydrolase and also produces thiosulfate. The simultaneous accumulation increase of sulfite and sulfide decreased the transcription of *cysI*, which encodes SiR for converting sulfite to sulfide.

### Sulfur metabolism changed by the overexpression of SOR

Overexpression of SOR in the recombinant accelerated the oxidation of S^0^ to RISCs, and ultimately ${\text{SO}}_{4}^{2-}$, after the time point at which the curves crossed. Accumulation increase of RISCs and ${\text{SO}}_{4}^{2-}$ in the recombinant culture resulted in the higher ORP and lower pH-values compared with the wild type. It is not an efficient way to produce electrons from sulfur atoms oxidized by SOR when sulfur atoms in cytoplasm are insufficient due to delay in sulfur activation and transport at the early stage of growth in S^0^-containing medium. At the early growth stage (before the time point at which the curves cross) in S^0^-containing medium, occupation of sulfur atoms induced by overexpression of SOR in the recombinant made shifting of the sulfur oxidation pathways from SOR to other efficient pathways impossible. In contrast, when sulfur atoms are sufficient at the late growth stage in the presence of S^0^, SOR overexpression in the recombinant oxidizes the sulfur atoms to produce other sulfur compounds, which enter other cytoplasmic sulfur oxidation pathways to produce electrons. This might be the reason for the ${\text{SO}}_{4}^{2--}$ accumulation increase, higher ORP, and lower pH of the SOR-overexpressing recombinant compared with the wild type in the late stage of growth. This hypothesis is also supported by the description of an obvious growth increase in a Δ*sor* mutant of *A. caldus* MH-04 observed in the first 6 (of a total of 12) days of culture in S^0^-containing medium (Quatrini et al., 2009). Sulfate plays an important role in the direct leaching of metals from mineral ores: MS + H_2_SO_4_ + 1/2 O^2^ → MSO_4_ + S^0^ + H_2_O, where M is a divalent metal (Suzuki, 2001). Leaching microorganisms will backfill the consumption of sulfate: S^0^ + 1/2 O^2^ + H_2_O → H_2_SO_4_ (Suzuki, 2001). Generally, lower pH will facilitate the sphalerite leaching (Mousavi et al., 2008; Vilcáez et al., 2009). The increased sulfate production in the SOR-overexpression strain indicated its potential metals leaching advantage via the direct action.

### Identification of tetrathionate reductase (Ttr)

The ability to respire tetrathionate based on *ttr* operon is characteristic of certain genera of *Enterobacteriaceae* such as *Salmonella typhimurium* (Hensel et al., 1999). Compared with TtrC, protein encoded by TPY_3700 showed significant sequence similarity to polysulfide reductase subunit C (PsrC). However, bioinformatics analysis indicated that the protein encoded by TPY_3700 owns typical conserved domain of TtrC. Hensel et al. also indicated that TtrC exhibits statistically significant sequence similarity to PsrC (Hensel et al., 1999). In addition, *ttrA, ttrB*, and TPY_3700 are clustered together and orientate in the same direction. As a result, TPY_3700 was predicted to be *ttrC*. According to bioinformatics analysis, TPY_3702 was predicted to be *ttrD*. The blast results of TtrD a.a. sequence from NCBI (National Center for Biotechnology Information) showed a conserved domain of TorD. Besides, protein-protein interaction databases of UniProt also showed that TtrD interacts with TtrA. TtrD belongs to the TorD/DmsD family chaperone, and binds specifically to the Tat signal peptide of the TtrA (Coulthurst et al., 2012).

## Conclusions

*Sulfobacillus acidophilus* TPY is a moderately thermoacidophilic Gram-positive bacterium with potential advantages in bioleaching. In this study, the sulfur metabolism pathways of *S. acidophilus* TPY with redox cycles were proposed via comparative transcriptomic analyses and RT-qPCR experiments. In order to confirm the sulfur metabolism pathways, SOR overexpression in *S. acidophilus* TPY and subsequent mRNA abundance analyses of sulfur metabolism related genes were carried out. The recombinant *S. acidophilus* TPY-SOR had higher sulfur metabolism activity resulting in ${\text{SO}}_{4}^{2-}$-accumulation increase, higher oxidation/reduction potentials (ORPs) and lower pH than the wild type in the late growth stage of S^0^ culture condition. The transcribed profile of *sor* gene and other sulfur metabolism related genes in both S^0^ and FeSO_4_ culture conditions confirmed the proposed sulfur metabolism pathways. This is the first attempt to characterize the sulfur metabolism pathways of Gram-positive *S. acidophilus* and also the first report of genetic manipulation of this species of Gram-positive moderate thermoacidophile.

## Availability of data and materials

The genome sequence of *S. acidophilus* TPY was deposited on GenBank with accession number of CP002901. The RNA-Seq reads have been deposited in GenBank with accession number SRP055734.

## Author contributions

All authors have read and approved the manuscript. WG and XC Design, acquisition of data, analysis and interpretation of data, drafting and revising the manuscript. HJZ, WZ, YW, and HBZ Acquisition of data, interpretation of data, revising the manuscript.

### Conflict of interest statement

The authors declare that the research was conducted in the absence of any commercial or financial relationships that could be construed as a potential conflict of interest.

## Abbreviations

SQR: sulfide quinone reductase
TQR: thiosulfate quinone reductase
TetH: tetrathionate reductase
TST: thiosulfate sulfur transferase (rhodanese)
HDR: heterodisulfide reductase
APS reductase: adenosine phosphosulfate reductase
SAT: sulfate adenylate transferase (sulfate adenylyltransferase, ATP sulfurylase)
SiR: sulfite reductase
Ttr: tetrathionate reductase
SOR: sulfur oxygenase reductase
Psr: polysulfide reductase
ORP: oxidation/ reduction potential
RISCs: reduced inorganic sulfur compounds
LB: Luria-Bertani
RPKM: reads per kb per million reads
FDR: false discovery rate
PVDF: polyvinylidene difluoride
QH_2_: quinone
SRB: sulfate reducing bacteria.

## References

[B1] Acevedo F. (2000). The use of reactors in biomining processes. Electron. J. Biotechnol. 3, 184–194. 10.2225/vol3-issue3-fulltext-4

[B2] Agterdenbos J. Martinius N. (1964). Theoretical considerations on the indirect determination of anions: determination of sulphate with barium chloranilate. Talanta 11, 875–885. 10.1016/0039-9140(64)80115-6

[B3] Amann E. Ochs B. Abel K. J. (1988). Tightly regulated tac promoter vectors useful for the expression of unfused and fused proteins in *Escherichia coli*. Gene 69, 301–315. 10.1016/0378-1119(88)90440-4 3069586

[B4] Audic S. Claverie J. M. (1997). The significance of digital gene expression profiles. Genome Res. 7, 986–995. 9331369 10.1101/gr.7.10.986

[B5] Auernik K. S. Maezato Y. Blum P. H. Kelly R. M. (2008). The genome sequence of the metal-mobilizing, extremely thermoacidophilic archaeon *Metallosphaera sedula* provides insights into bioleaching-associated metabolism. Appl. Environ. Microbiol. 74, 682–692. 10.1128/AEM.02019-07 18083856 PMC2227735

[B6] Berks B. C. Sargent F. Palmer T. (2000). The Tat protein export pathway. Mol. Microbiol. 35, 260–274. 10.1046/j.1365-2958.2000.01719.x 10652088

[B7] Brierley J. A. (1990). Acdidophilic thermophilic archaebacteria: potential application for metals recovery. FEMS Microbiol. Lett. 75, 287–291. 10.1111/j.1574-6968.1990.tb04102.x

[B8] Brierley J. A. Brierley C. L. (2001). Present and future commercial applications of biohydrometallurgy. Hydrometallurgy 59, 233–239. 10.1016/S0304-386X(00)00162-6

[B9] Brune K. D. Bayer T. (2012). Engineering microbial consortia to enhance biomining and bioremediation. Front. Microbiol. 3:203. 10.3389/fmicb.2012.00203 22679443 PMC3367458

[B10] Chen L. Ren Y. Lin J. Liu X. Pang X. Lin J. (2012). *Acidithiobacillus caldus* sulfur oxidation model based on transcriptome analysis between the wild type and sulfur oxygenase reductase defective mutant. PLoS ONE 7:e39470. 10.1371/journal.pone.0039470 22984393 PMC3440390

[B11] Coulthurst S. J. Dawson A. Hunter W. N. Sargent F. (2012). Conserved signal peptide recognition systems across the prokaryotic domains. Biochemistry 51, 1678–1686. 10.1021/bi201852d 22289056 PMC3290102

[B12] Dahl C. Franz B. Hensen D. Kesselheim A. Zigann R. (2013). Sulfite oxidation in the purple sulfur bacterium *Allochromatium vinosum*: identification of SoeABC as a major player and relevance of SoxYZ in the process. Microbiology 159, 2626–2638. 10.1099/mic.0.071019-0 24030319

[B13] De Jong Gah H. W. Bos P. Kuenen J. G. (1997). Polythionate degradation by tetrathionate hydrolase of *Thiobacillus ferrooxidans*. Microbiology 143, 499–504. 10.1099/00221287-143-2-499 33711857

[B14] Dopson M. Johnson D. B. (2012). Biodiversity, metabolism and applications of acidophilic sulfur-metabolizing microorganisms. Environ. Microbiol. 14, 2620–2631. 10.1111/j.1462-2920.2012.02749.x 22510111

[B15] Ghosh W. Dam B. (2009). Biochemistry and molecular biology of lithotrophic sulfur oxidation by taxonomically and ecologically diverse bacteria and archaea. FEMS Microbiol. Rev. 33, 999–1043. 10.1111/j.1574-6976.2009.00187.x 19645821

[B16] Guo X. Yin H. Liang Y. Hu Q. Zhou X. Xiao Y.. (2014). Comparative genome analysis reveals metabolic versatility and environmental adaptations of *Sulfobacillus thermosulfidooxidans* strain ST. PLoS ONE 9:e99417. 10.1371/journal.pone.0099417 24940621 PMC4062416

[B17] Hensel M. Hinsley A. P. Nikolaus T. Sawers G. Berks B. C. (1999). The genetic basis of tetrathionate respiration in *Salmonella typhimurium*. Mol. Microbiol. 32, 275–287. 10.1046/j.1365-2958.1999.01345.x 10231485

[B18] Hoang T. T. Karkhoff-Schweizer R. R. Kutchma A. J. Schweizer H. P. (1998). A broad-host-range Flp-FRT recombination system for site-specific excision of chromosomally-located DNA sequences: application for isolation of unmarked *Pseudomonas aeruginosa* mutants. Gene 212, 77–86. 10.1016/S0378-1119(98)00130-9 9661666

[B19] Johnson D. B. Kanao T. Hedrich S. (2012). Redox transformations of iron at extremely low pH: fundamental and applied aspects. Front. Microbiol. 3:96. 10.3389/fmicb.2012.00096 22438853 PMC3305923

[B20] Joubert T. M. (2008). Towards a Genetic System for the Genus Sulfobacillus. Master's Thesis, University of Stellenbosch.

[B21] Justice N. B. Norman A. Brown C. T. Singh A. Thomas B. C. Banfield J. F. (2014). Comparison of environmental and isolate Sulfobacillus genomes reveals diverse carbon, sulfur, nitrogen, and hydrogen metabolisms. BMC Genomics 15:1107. 10.1186/1471-2164-15-1107 25511286 PMC4378227

[B22] Kjeldsen K. U. Joulian C. Ingvorsen K. (2004). Oxygen tolerance of sulfate-reducing bacteria in activated sludge. Environ. Sci. Technol. 38, 2038–2043. 10.1021/es034777e 15112804

[B23] Kletzin A. Urich T. Müller F. Bandeiras T. M. Gomes C. M. (2004). Dissimilatory oxidation and reduction of elemental sulfur in thermophilic archaea. J. Bioenerg. Biomembr. 36, 77–91. 10.1023/B:JOBB.0000019600.36757.8c 15168612

[B24] Li B. Chen Y. Liu Q. Hu S. Chen X. (2011). Complete genome analysis of *Sulfobacillus acidophilus* strain TPY, isolated from a hydrothermal vent in the Pacific Ocean. J. Bacteriol. 193, 5555–5556. 10.1128/JB.05684-11 21914875 PMC3187392

[B25] Li R. Li Y. Fang X. Yang H. Wang J. Kristiansen K.. (2009). SNP detection for massively parallel whole-genome resequencing. Genome Res. 19, 1124–1132. 10.1101/gr.088013.108 19420381 PMC2694485

[B26] Liu Y. Lin-Wang K. Deng C. Warran B. Wang L. Yu B. (2015). Comparative transcriptome analysis of white and purple potato to identify genes involved in anthocyanin biosynthesis. PLoS ONE 10:e0129148 10.1145/2818302 26053878 PMC4459980

[B27] Livak K. J. Schmittgen T. D. (2001). Analysis of relative gene expression data using real-time quantitative PCR and the 2(-Delta Delta C(T)) Method. Methods 25, 402–408. 10.1006/meth.2001.1262 11846609

[B28] Mangold S. Valdés J. Holmes D. Dopson M. (2011). Sulfur metabolism in the extreme acidophile *Acidithiobacillus caldus*. Front. Microbiol. 2:17. 10.3389/fmicb.2011.00017 21687411 PMC3109338

[B29] Merroun M. L. Geipel G. Nicolai R. Heise K. H. Selenska-Pobell S. (2003). Complexation of uranium (VI) by three eco-types of *Acidithiobacillus ferrooxidans* studied using time-resolved laser-induced fluorescence spectroscopy and infrared spectroscopy. Biometals 16, 331–339. 10.1023/A:1020612600726 12572691

[B30] Mihara H. Esaki N. (2002). Bacterial cysteine desulfurases: their function and mechanisms. Appl. Microbiol. Biotechnol. 60, 12–23. 10.1007/s00253-002-1107-4 12382038

[B31] Mortazavi A. Williams B. A. Mccue K. Schaeffer L. Wold B. (2008). Mapping and quantifying mammalian transcriptomes by RNA-Seq. Nat. Methods 5, 621–628. 10.1038/nmeth.1226 18516045 PMC13303166

[B32] Mousavi S. M. Yaghmaei S. Vossoughi M. Roostaazad R. Jafari A. Ebrahimi M.. (2008). The effects of Fe(II) and Fe(III) concentration and initial pH on microbial leaching of low-grade sphalerite ore in a column reactor. Bioresour. Technol. 99, 2840–2845. 10.1016/j.biortech.2007.06.009 17698352

[B33] Murphy M. J. Siegel L. M. Tove S. R. Kamin H. (1974). Siroheme: a new prosthetic group participating in six-electron reduction reactions catalyzed by both sulfite and nitrite reductases. Proc. Natl. Acad. Sci. U.S.A. 71, 612–616. 10.1073/pnas.71.3.612 4595566 PMC388061

[B34] Norris P. R. Clark D. A. Owen J. P. Waterhouse S. (1996). Characteristics of *Sulfobacillus acidophilus* sp. nov. and other moderately thermophilic mineral-sulphide-oxidizing bacteria. Microbiology 142(Pt 4), 775–783. 10.1099/00221287-142-4-775 8936305

[B35] Olson G. J. Brierley J. A. Brierley C. L. (2003). Bioleaching review part B: progress in bioleaching: applications of microbial processes by the minerals industries. Appl. Microbiol. Biotechnol. 63, 249–257. 10.1007/s00253-003-1404-6 14566430

[B36] Peng J. B. Yan W. M. Bao X. Z. (1994). Plasmid and transposon transfer to *Thiobacillus ferrooxidans*. J. Bacteriol. 176, 2892–2897. 8188590 10.1128/jb.176.10.2892-2897.1994 PMC205444

[B37] Qin N. Tan X. Jiao Y. Liu L. Zhao W. Yang S.. (2014). RNA-Seq-based transcriptome analysis of methicillin-resistant *Staphylococcus aureus* biofilm inhibition by ursolic acid and resveratrol. Sci. Rep. 4:5467. 10.1038/srep05467 24970710 PMC4073122

[B38] Quatrini R. Appia-Ayme C. Denis Y. Jedlicki E. Holmes D. S. Bonnefoy V. (2009). Extending the models for iron and sulfur oxidation in the extreme acidophile *Acidithiobacillus ferrooxidans*. BMC Genomics 10:394. 10.1186/1471-2164-10-394 19703284 PMC2754497

[B39] Rawlings D. E. (2002). Heavy metal mining using microbes. Annu. Rev. Microbiol. 56, 65–91. 10.1146/annurev.micro.56.012302.161052 12142493

[B40] Rawlings D. E. Dew D. Du Plessis C. (2003). Biomineralization of metal-containing ores and concentrates. Trends Biotechnol. 21, 38–44. 10.1016/S0167-7799(02)00004-5 12480349

[B41] Robertson W. J. Kinnunen P. H. M. Plumb J. J. Franzmann P. D. Puhakka J. A. Gibson J. (2002). Moderately thermophilic iron oxidising bacteria isolated from a pyritic coal deposit showing spontaneous combustion. Miner. Eng. 15, 815–822. 10.1016/S0892-6875(02)00130-9

[B42] Simon R. Priefer U. Puhler A. (1983). A broad host range mobilization system for *in vivo* genetic engineering: transposon mutagenesis in gram negative bacteria. Nat. Biotech. 1, 784–791. 10.1038/nbt1183-784

[B43] Suzuki I. (2001). Microbial leaching of metals from sulfide minerals. Biotechnol. Adv. 19, 119–132. 10.1016/S0734-9750(01)00053-2 14538087

[B44] Thomas D. Surdin-Kerjan Y. (1997). Metabolism of sulfur amino acids in *Saccharomyces cerevisiae*. Microbiol. Mol. Biol. Rev. 61, 503–532. 9409150 10.1128/mmbr.61.4.503-532.1997 PMC232622

[B45] Torma A. E. (1983). Biotechnology applied to mining of metals. Biotechnol. Adv. 1, 73–80. 10.1016/0734-9750(83)90302-6 14544247

[B46] Valdes J. Pedroso I. Quatrini R. Dodson R. J. Tettelin H. Blake R. II.. (2008). *Acidithiobacillus ferrooxidans* metabolism: from genome sequence to industrial applications. BMC Genomics 9:597. 10.1186/1471-2164-9-597 19077236 PMC2621215

[B47] Vilcáez J. Yamada R. Inoue C. (2009). Effect of pH reduction and ferric ion addition on the leaching of chalcopyrite at thermophilic temperatures. Hydrometallurgy 96, 62–71. 10.1016/j.hydromet.2008.08.003

[B48] Wang A. Ren N. Wang X. Lee D. (2008). Enhanced sulfate reduction with acidogenic sulfate-reducing bacteria. J. Hazard. Mater. 154, 1060–1065. 10.1016/j.jhazmat.2007.11.022 18093734

[B49] Zeghouf M. Fontecave M. Coves J. (2000). A simplifed functional version of the *Escherichia coli* sulfite reductase. J. Biol. Chem. 275, 37651–37656. 10.1074/jbc.M005619200 10984484

[B50] Zeng J. Wang M. Zhang X. Wang Y. Ai C. Liu J.. (2008). Expression, purification and characterization of the sulfite reductase hemo-subunit, SiR-HP, from *Acidithiobacillus ferrooxidans*. Biotechnol. Lett. 30, 1239–1244. 10.1007/s10529-008-9679-4 18317695

[B51] Zhang H. Guo W. Xu C. Zhou H. Chen X. (2013). Site-specific mutagenesis and functional analysis of active sites of sulfur oxygenase reductase from Gram-positive moderate thermophile *Sulfobacillus acidophilus* TPY. Microbiol. Res. 168, 654–660. 10.1016/j.micres.2013.04.008 23726793

[B52] Zhou H.-B. Zeng W.-M. Yang Z.-F. Xie Y.-J. Qiu G.-Z. (2009). Bioleaching of chalcopyrite concentrate by a moderately thermophilic culture in a stirred tank reactor. Bioresour. Technol. 100, 515–520. 10.1016/j.biortech.2008.06.033 18657418

