# Supplementary material for: Sulfur Metabolism Pathways in Sulfobacillus acidophilus TPY, A Gram-Positive Moderate Thermoacidophile from a Hydrothermal Vent
Source: Front Microbiol. 2016 Nov 18;7:1861. doi: 10.3389/fmicb.2016.01861 (PMC5114278; doi:10.3389/fmicb.2016.01861)
Supplement: Supplementary file 1 [file Table1.DOC]

**Table 1 Strains and** plasmids used in this study

| Strain/ plasmid | Relevant characteristics | Source or reference |
| --- | --- | --- |
| *E.coli* |  |  |
| JM109 | recA1 endA1 gyrA96 thi-1 hsdR17 supE44 relA1 D(lac-proAB) | Stratagene |
| S17-1 | recA; harbors the tra genes of plasmid RP4 in the chromosome; proA thi-1 | [21] |
| *S. acidophilus* |  |  |
| TPY | gram-positive，thermoacidophilic; Cm^R^ ^a^ | This study |
| TPY-SOR | TPY derivative harboring plasmid pTrc99A_sor_oriT; Cm^R^; Amp^R b^ | This study |
| Plasmids |  |  |
| pEX18Tc | Tc^R^; oriT^+^ sacB^+^, gene replacement vector with MCS from pUC18 ^c^ | [31] |
| pTrc99A | Shuttle vector of *E. coli* and *Bacillus*; Amp^R^ | [30] |
| pTrc99A_sor | pTrc99A derivative containing *sor* gene from *S. acidophilus* TPY genome; Amp^R^ | This study |
| pTrc99A_sor_oriT | pTrc99A_sor derivative containing oriT gene from pEX18Tc; Amp^R^ | This study |

^a^ Cm, chloramphenicol; ^b^ Amp, ampicillin; ^c^ Tc, tetracycline; ^R^ resistant.
