# Supplementary material for: Sulfur Metabolism Pathways in Sulfobacillus acidophilus TPY, A Gram-Positive Moderate Thermoacidophile from a Hydrothermal Vent
Source: Front Microbiol. 2016 Nov 18;7:1861. doi: 10.3389/fmicb.2016.01861 (PMC5114278; doi:10.3389/fmicb.2016.01861)
Supplement: Supplementary file 2 [file Table2.DOC]

**Table 2 The list of primer**s used in this study

| Primers | Sequences | Sources |
| --- | --- | --- |
| Gene expression |  |  |
| PTrc99A_sor_fwd ^a^ | 5’-CG*GAATTC*CTGGAGATGAAGATACCTATGCCCGTACCGTACA T | This study |
| pTrc99A_sor_rev ^b^ | 5’-CG*GGATCC*TTAGTGATGA TGATGATGATGTGAGTGCAAATACTCTC | This study |
| pTrc99A_oriT_fwd | 5’-CG*GGATCC*CTAGAGTCGATCTTCGCCAG | This study |
| pTrc99A_oriT_rev | 5’-CC*AAGCTT* CGCCTGATGCGGTATTTTCTCCTTAC | This study |
| Real time PCR |  |  |
| 16SrRNA_fwd | 5’-CCTTCAAACGGCGTCTCAG | This study |
| 16SrRNA_rev | 5'-TTACGACTTCACCCCAATCATC | This study |
| TPY_0405_fwd | 5’-GTTAGGAGCGGCGTGGAT | This study |
| TPY_0405_rev | 5'-GGTTCCATAGTGTCACATAAGG | This study |
| TPY_2303_fwd | 5’-CACTTGTCATTCATCCTCTTGTTGGTCC | This study |
| TPY_2303_rev | 5’-GAAATAACGCTTCCCGAGGTCCG | This study |
| TPY_2304_fwd | 5’-CGAGAGCAAAGCCCGACACGA | This study |
| TPY_2304_rev | 5’-GAACTTATCCCATCCTTCCCATCGTC | This study |
| TPY_2305_fwd | 5’-CGCCGCAACATCATTTACTAC | This study |
| TPY_2305_rev | 5’-GTCTCGATTTGAGCCACCAC | This study |
| TPY_0895_fwd | 5’-GGTCGTCTATGTGGGGAG | This study |
| TPY_0895_rev | 5’-TGTATTGGCTGATTTCTTGC | This study |
| TPY_2169_fwd | 5’-GACTGGTACTCCAAGCATAA | This study |
| TPY_2169_rev | 5’-CCTTCCACTGAACCGATT | This study |
| TPY_0364_fwd | 5’-TCGGAGGAATGGAATACAC | This study |
| TPY_0364_rev | 5’-TCGTCAGAGTCGTATAGGT | This study |
| TPY_3700_fwd | 5’-GAGCCTATTCGTTCTGATTAG | This study |
| TPY_3700_rev | 5’-GTAATGAGGAGAAGAGCAATG | This study |
| TPY_0115_fwd | 5’-CGATGCTATATGCCACGAT | This study |
| TPY_0115_rev | 5’-GCTAACAGGACCGATGTC | This study |
| TPY_3079_fwd | 5’-CAATGCTAAGGTGGTCAATG | This study |
| TPY_3079_rev | 5’-ACCGCCGAATGGATAATAC | This study |

^a^ the italic indicate the restriction endonuclease cutting sites; ^b^ the underline indicate the 6 ×His tag encoding region.
