# Supplementary figures and images for: Sulfur Metabolism Pathways in Sulfobacillus acidophilus TPY, A Gram-Positive Moderate Thermoacidophile from a Hydrothermal Vent
Source: Front Microbiol. 2016 Nov 18;7:1861. doi: 10.3389/fmicb.2016.01861 (PMC5114278; doi:10.3389/fmicb.2016.01861)

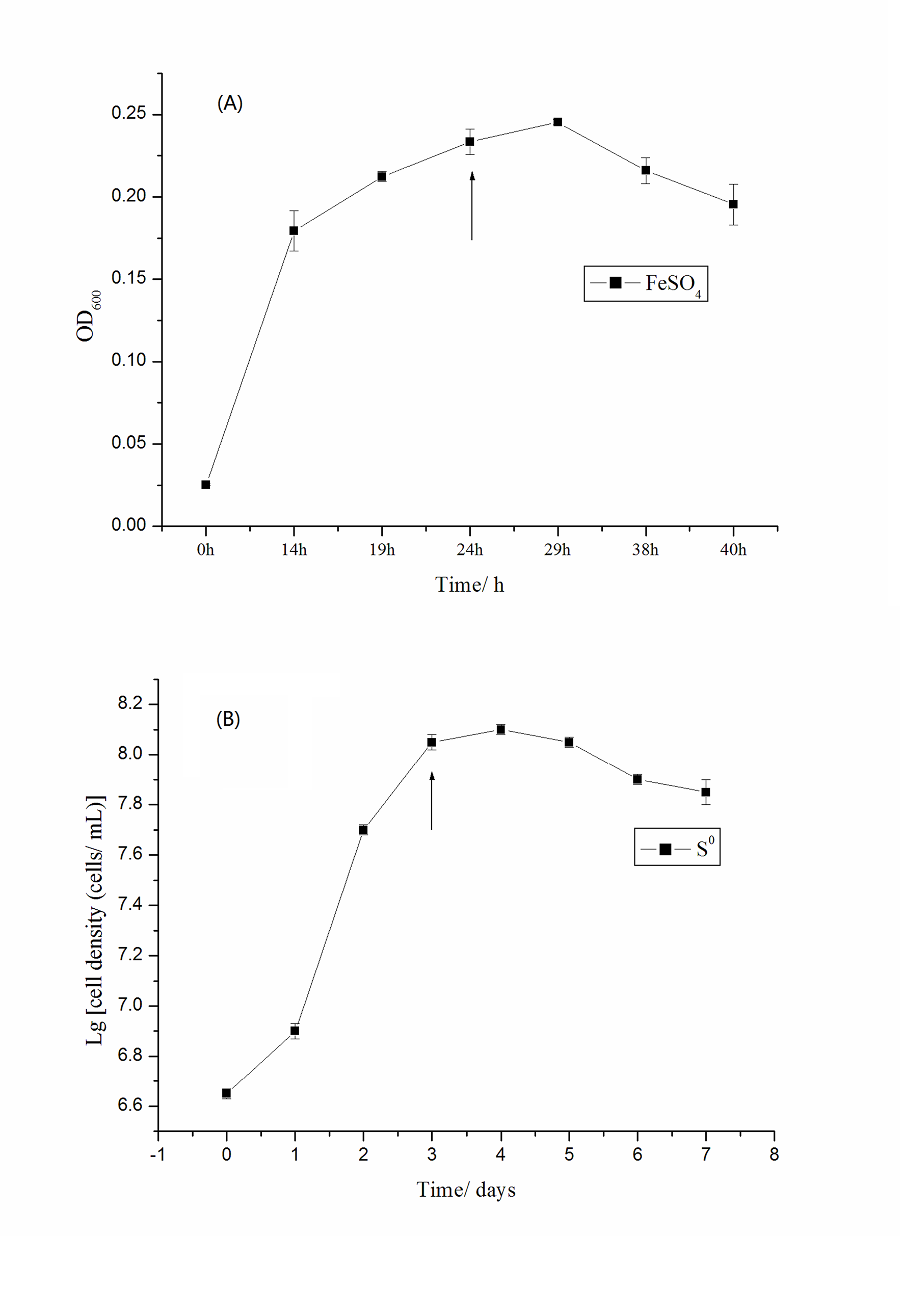

Supplement: Supplementary file 4 [file Image1.TIF]

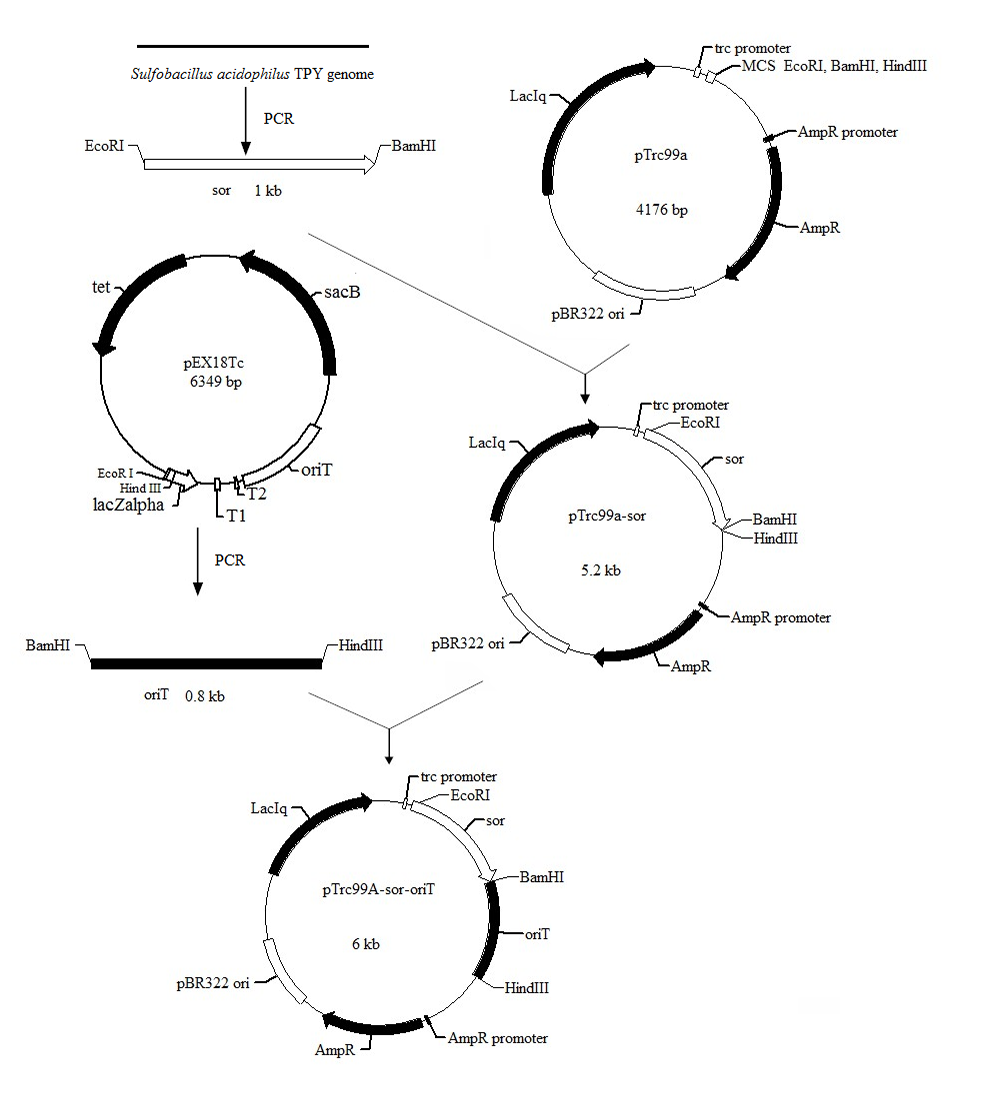

Supplement: Supplementary file 5 [file Image2.TIF]
